# Clinical value of histologic endometrial dating for personalized frozen-thawed embryo transfer in patients with repeated implantation failure in natural cycles

**DOI:** 10.1186/s12884-020-03217-y

**Published:** 2020-09-11

**Authors:** Yuan Li, Xiao feng Li, Jing nan Liao, Xiang xiu Fan, Yong bin Hu, Runxin Gan, Guangxiu Lu, Ge Lin, Fei Gong

**Affiliations:** 1 Institute of Reproduction and Stem Cell Engineering, Basic Medicine College, Central South University, Changsha, China; 2 Reproductive and Genetic Hospital of CITIC-XIANGYA, Changsha, China; 3 Key Laboratory of Stem Cells and Reproductive Engineering, National Health and Family Planning Commission, Changsha, China; 4 Department of Pathology, Xiang Ya Hospital, Central South University, Changsha, 410008 China

**Keywords:** Histologic endometrial dating, Endometrial receptivity, Personal embryo transfer, Window of implantation

## Abstract

**Background:**

Displacement of the window of implantation (WOI) has been proposed as an important factor contributing to repeated implantation failure (RIF). However, the use of histologic endometrial dating as a diagnostic tool of endometrial receptivity has been questioned.

**Methods:**

This study is a prospective intervention trial that enrolled 205 infertile patients from July 2017 to December 2017. Endometrial biopsies from 50 patients with good prognoses were conducted on day 3 (*n* = 6), 5 (n = 6), 7 (*n* = 26), 9 (n = 6), or 11 (n = 6) post-ovulation (PO + 3/5/7/9/11) of the previous natural cycle before their conventional frozen-thawed embryo transfer (FET) cycle. We conducted endometrial biopsies for 155 RIF patients on day PO + 7.

**Results:**

The verification of the Noyes criteria for endometrial dating was conducted at different times (PO + 3/+ 5/+ 7/+ 9/+ 11) on 41 patients with good prognoses who achieved an ongoing pregnancy in their first conventional FET cycle after endometrial biopsy. The agreement between two pathologists determining endometrial biopsy dating results in infertile patients was determined to be acceptable (weighted kappa = 0.672, *P* < 0.001). The rate of out-of-phase dating on day PO + 7 was significantly higher in RIF patients than in good- prognosis patients (31.6% vs. 3.8%, *P* = 0.003). pFET was performed in 47 RIF patients diagnosed to be out of phase, and the cumulative live-birth rate was 61.7%.

**Conclusions:**

Histologic endometrial dating of RIF patients in natural cycles may be a biomarker for a receptive endometrium in diagnosing WOI displacement.

**Trial registration:**

NCT03312309 Registered 17 October 2017*.*

NCT03222830 Registered 19 July 2017,

## Background

Repeated implantation failure (RIF) is a particular challenge that is defined as the absence of a gestational sac at five or more weeks after an embryo transfer (ET) subsequent to three previous embryo transfers with high-quality embryos, or after the transfer of ≥10 embryos in multiple transfers [1]. RIF can be caused by both maternal and embryonic factors [2], and blastocyst culture and preimplantation genetic screening can partially improve pregnancy outcome through better embryo selection [3]. The uterus, an important player in implantation, may be affected by polyps, intrauterine adhesions, uterine fibroids, adenomyosis, endometritis, and uterine malformations, and has been demonstrated to contribute to embryonic implantation failure [4]. Different strategies have been developed to improve pregnancy outcomes for the aforementioned diseases, but unexplained RIF remains a challenge.

Endometrial receptivity has been frequently evaluated as one of the many uterine factors involved in RIF, but the relationship remains controversial. Several endometrial markers, such as the presence of pinopods, immunohistochemical biomarkers, and endometrial waves and blood flow, have been used to determine uterine receptivity. These biomarkers may interact transiently with the embryo at implantation, but they appear to be unreliable for evaluating receptivity, particularly as precision indicators for use as clinical diagnostic tools [5–7].

The endometrium becomes receptive to implantation as a result of a series of timed hormonal events during the menstrual cycle. Endometrial exposure to progesterone after ovulation initiates morphological and functional alterations, triggering a shift from a pre-receptive state to a receptive state. With the secretion of progesterone, subnuclear vacuoles, which are found in epithelial cells during the early secretory phase, discharge the secretory products within their glandular epithelium cells to the glandular lumen, and stromal edema becomes maximal in the middle secretory phase, all of which contributes to blastocyst adhesion and invasion. In this phase, edema is also less marked, and a predecidual reaction begins around the blood vessels, contributing to embryonic implantation. The morphological changes observed histologically for each specific day after ovulation were described by Noyes and his colleagues in 1950, and termed the Noyes criteria [8]. An endometrial biopsy that shows a difference of more than 2 days between the histologic dating and actual day after ovulation is considered to be “out of phase” [9]. However, the clinical application of the Noyes criteria is relatively limited, as an out-of-phase endometrium has also been found in 5–50% of fertile patients [10–12]. The large variation in researchers’ results may be due to inaccurate determination of the ovulation day.

Previous investigators demonstrated that classic histologic dating of endometrial biopsy samples could be used to estimate the timing of the window of implantation and to adjust embryonic transfer time [13]. This in turn could potentially increase the implantation rate of patients with an out-of-phase endometrium during hormone replacement therapy (HRT) cycles [13]. The clinical value of histologic endometrial dating in RIF patients during natural cycles, however, has yet to be determined. In the present study, we investigated the clinical effects of personalized frozen-thawed embryo transfer (pFET) in patients with unexplained RIF using histologic dating of endometrial biopsies, which were performed under ultrasound-guided ovulation monitoring during natural cycles.

## Methods

### Study population

**Fig. 1:**
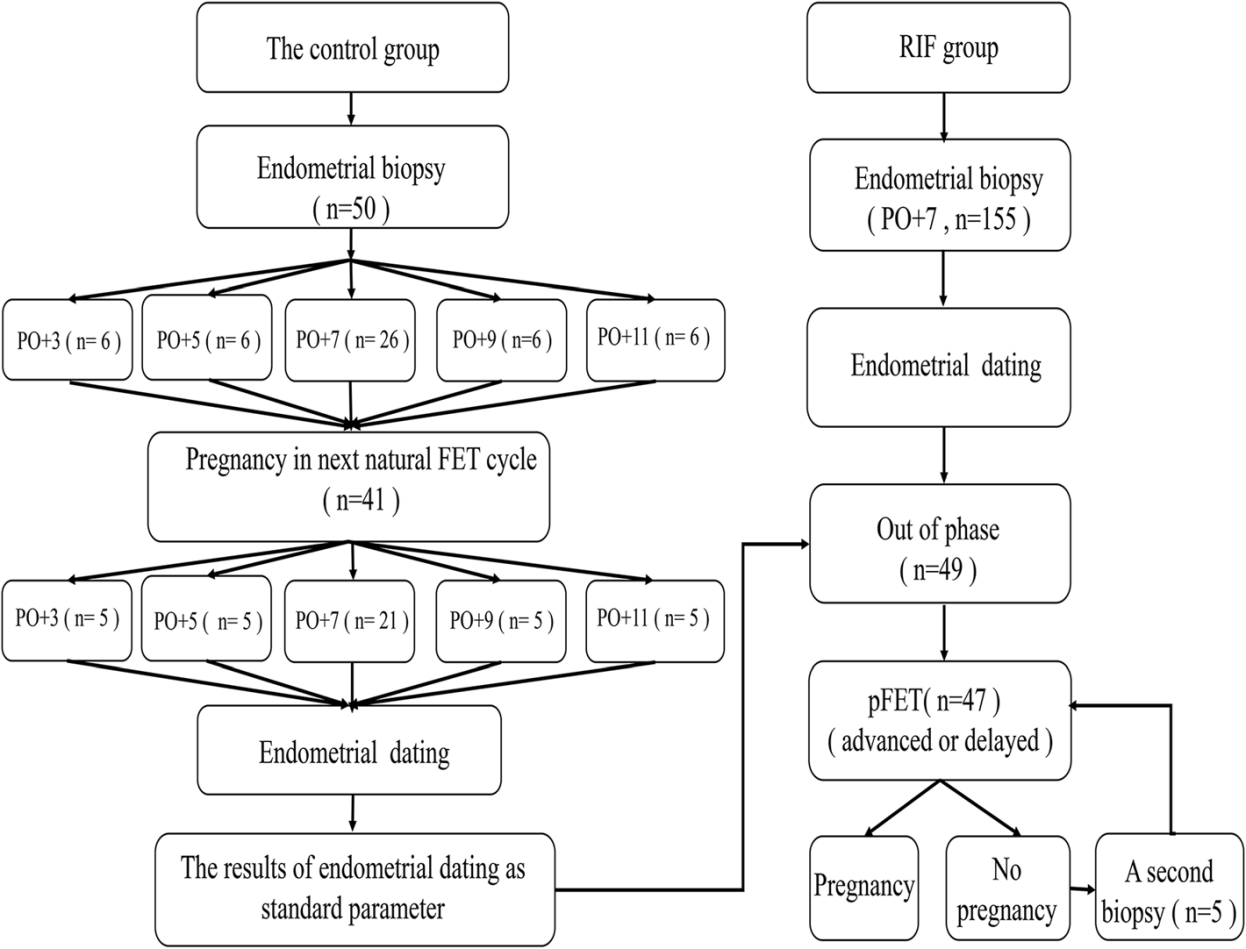
The patients recruited to the control and RIF groups

In this pilot study, we evaluated a total of 205 infertile patients and created two phases. In phase I, a total of 50 patients with good prognoses underwent endometrial biopsy at different time-points (PO + 3/5/7/9/11) (Fig. 1). The histological profiles of good-prognosis patients who were pregnant in their first conventional FET cycle were then collected as fertility parameters. For the good-prognosis patient group, we enrolled women aged 20–35 who underwent FET in a natural cycle. In phase II, 155 patients with unexplained RIF were recruited for endometrial dating evaluation on PO + 7 (Fig. 1). According to an ESHRE PGD consortium, RIF was defined as the absence of a gestational sac upon ultrasonographic examination at five or more weeks subsequent to three embryo transfers with high-quality embryos, or after the transfer of ≥10 embryos in multiple transfers [1]. Patients with uterine abnormalities (double uterus, bicornuate uterus, unicornuate uterus, and uterine mediastinum), intrauterine adhesions, endometriosis, adenomyosis, hydrosalpinx, or uterine fibroids (submucosal fibroids, non-mucosal fibroids > 4 cm and/or endometrial pressure) were excluded from the unexplained-RIF group. In both groups, patients demonstrated a menstrual cycle length of 24–35 days and an indication for ovarian stimulation before in vitro fertilization/intracytoplasmic sperm injection (IVF/ICSI). This study was approved by the Ethics Committee of the Reproductive and Genetic Hospital of CITIC-XIANGYA (LL-SC-2017-007) (June 29, 2017). Although we began to recruit the patients on the initial release date of our clinical trial, we discovered the advantages of histologic dating of endometrial biopsy samples when we designed and organized the data of the first clinical trial (NCT03222830). We then increased the sample size of our study, which was of major importance in recruiting RIF patients and good-prognosis patients for histologic dating for the second clinical trial (NCT03312309).

### Ovulation monitoring

All patients were monitored throughout a natural cycle, with a daily ultrasonographic scan from the 10th–12th days of the menstrual cycle when the largest follicular diameter was 16 mm, and until the dominant follicle disappeared. Urinary LH concentrations were assessed simultaneously when the follicular diameter was 16 mm. The day of dominant follicle disappearance was considered to be the day of ovulation (post-ovulation + 0, PO + 0).

### Endometrial biopsy

Endometrial biopsy was performed using a sterile pipelle (Laboratory CCD, China), and the tissue was stored in Hank’s Balanced Salt Solution (Life Technologies, Grand Island, NY) on ice for further processing.

### Histologic analysis and dating

Endometrial tissue was rinsed in chilled PBS, followed by fixation and paraffin embedding (FFPE) with 10% neutral-buffered formalin. FFPE tissues were sectioned at a 6 mm thickness for hematoxylin and eosin (H&E) staining. All H&E-stained endometrial biopsies were analyzed in a blinded manner to evaluate endometrial dating and glandular and stromal development. The endometrial dating was verified according to the Noyes dating criteria [8].

### Personal frozen embryo transfer/conventional frozen embryo transfer protocol

For the FET cycle, no more than two embryos were transferred to each patient. Embryos were warmed using a commercially available warming solution (Kitazato Biopharma), according to the Kuwayama kit instructions [14]. After warming, embryos were transferred to G1.5/G2.5 medium and cultured for 2–6 h. Only cleavage-stage embryos that exhibited > 50% intact blastomeres or blastocysts that re-expanded after warming were considered as surviving and suitable for transfer. For patients with good prognoses, the cleavage-stage embryos or blastocysts were transferred either 3 or 5 days, respectively, after ovulation, regardless of endometrial dating. For the RIF group, they were transferred 4–7 days after ovulation, depending upon the endometrial dating results. We applied luteal support when the dominant follicle disappeared, and when we observed satisfactory endometrial development (thickness ≥ 8 mm as confirmed by ultrasonographic examination), we administered 40 mg of dydrogesterone (Abbott Biologicals B.V.) until the 28th day of embryo transfer if a pregnancy test was positive.

### Clinical outcomes and statistical analysis

We defined the cumulative live-birth rate of repeated FET cycles during the study period as the probability of a live birth from all patients during the study period. Ongoing pregnancy was defined as at least one intrauterine gestational sac with cardiac activity by ultrasonography performed 6 weeks after ET. Biochemical pregnancy was defined as a positive hCG test in the absence of an intrauterine gestational sac. Analyses were performed using the statistical package SPSS, version 19.0 (SPSS) or (SAS®) version 9.3 (SAS Institute, Inc., Cary, NC, USA). Continuous variables are presented as the mean ± standard deviation (SD), and comparisons were made using a one-way ANOVA or non-parametric statistical tests. Categorical data are presented as a number (N) and percentage (%), and comparisons were made using a Chi-square or Fisher Exact Probability test. A weighted kappa statistic was calculated to summarize the overall agreement between pathologist A and pathologist B. Bland–Altman plots were drawn to evaluate systematic biases of endometrial dating between pathologists A and B. We used GraphPad Prism7.0 to evaluate the intra-group difference (mean ± SD) in the control group. *P* < 0.05 was considered to be statistically significant.

## Results

### Noyes criteria verification

Standard endometrial histologic dating parameters were established from the patients with good prognoses who were pregnant in their first conventional FET cycle (*n* = 41). All the good-prognosis patients with ongoing pregnancies included in this study went on to have a live birth. With respect to PO dating + 3, glandular nuclei were pushed to the center of the epithelial cells with cytoplasm above and vacuoles below. For PO dating + 4, glandular nuclei returned to the basilar side of the cell. We noted that wisps of secretory material appeared in the lumina, and some vacuoles were pushed past the nucleus, apparently emptying their glycogen into the lumen. Mitosis and pseudostratification of nuclei were absent. For PO dating + 5, only a few vacuoles remained. For PO dating + 7, tissue edema, although variable in the proliferative phase, was characteristically notable in the mid-secretory stage, becoming evident rather suddenly. For PO dating + 9, the spiral arterioles (which were previously somewhat difficult to distinguish in the edematous stroma) became much more prominent. For PO dating + 11, pre-decidua began to differentiate under the surface epithelium (Fig. S1). These results were consistent with the Noyes dating criteria [8].

### Endometrial dating criteria and blinded pathologist agreement

**Fig. 2:**
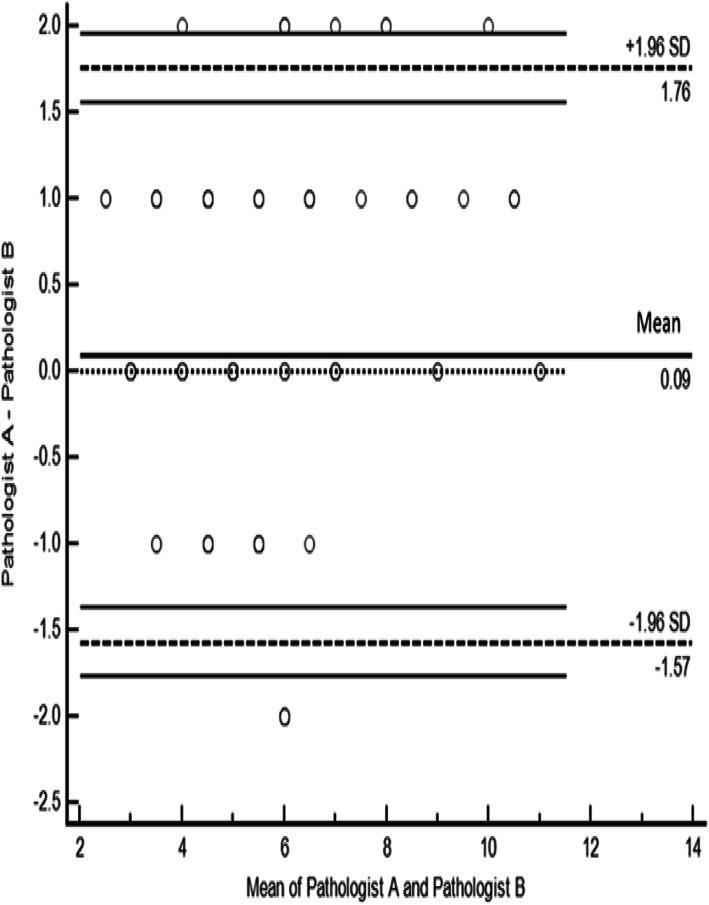
Bland–Altman plots of variability according to pathologists A and B. The x-axis depicts the mean endometrial sample dating for pathologist A and pathologist B; the y-axis is the difference from the mean endometrial dating between pathologists A and B. The upper and lower lines on the B–A plots represent the limits of agreement and the mean difference ± 1.96 times its standard deviation. Thus, the distance from 0 and the width of the limits of agreement both indicate the magnitude of disagreement between pathologists. Closer clustering to the mean indicates higher agreement. If the difference value for an endometrial dating is = 0, then the endometrial dating by pathologist A and pathologist B was identical

All endometrial dating (*n* = 205) was determined by two experienced pathologists. The inter-observer agreement was determined to be good (weighted kappa = 0.672; 95% CI 0.606–0.737; *P* < 0.001). As shown in Fig. 2, Bland-Altman (B-A) plots of pathologist A and pathologist B highlighted trends regarding differences in endometrial dating between the two pathologists. The limits of agreement indicated that the difference value for dating an endometrial biopsy was ≤1.76, but that the endometrial dating by both pathologists was clinically consistent when the value was ≤2. Thus, the B-A plots suggested that agreement was good between pathologists A and B when the value was ≤2 using the Noyes criteria in the same patients.

### Out-of-phase endometrial dating

**Fig. 3:**
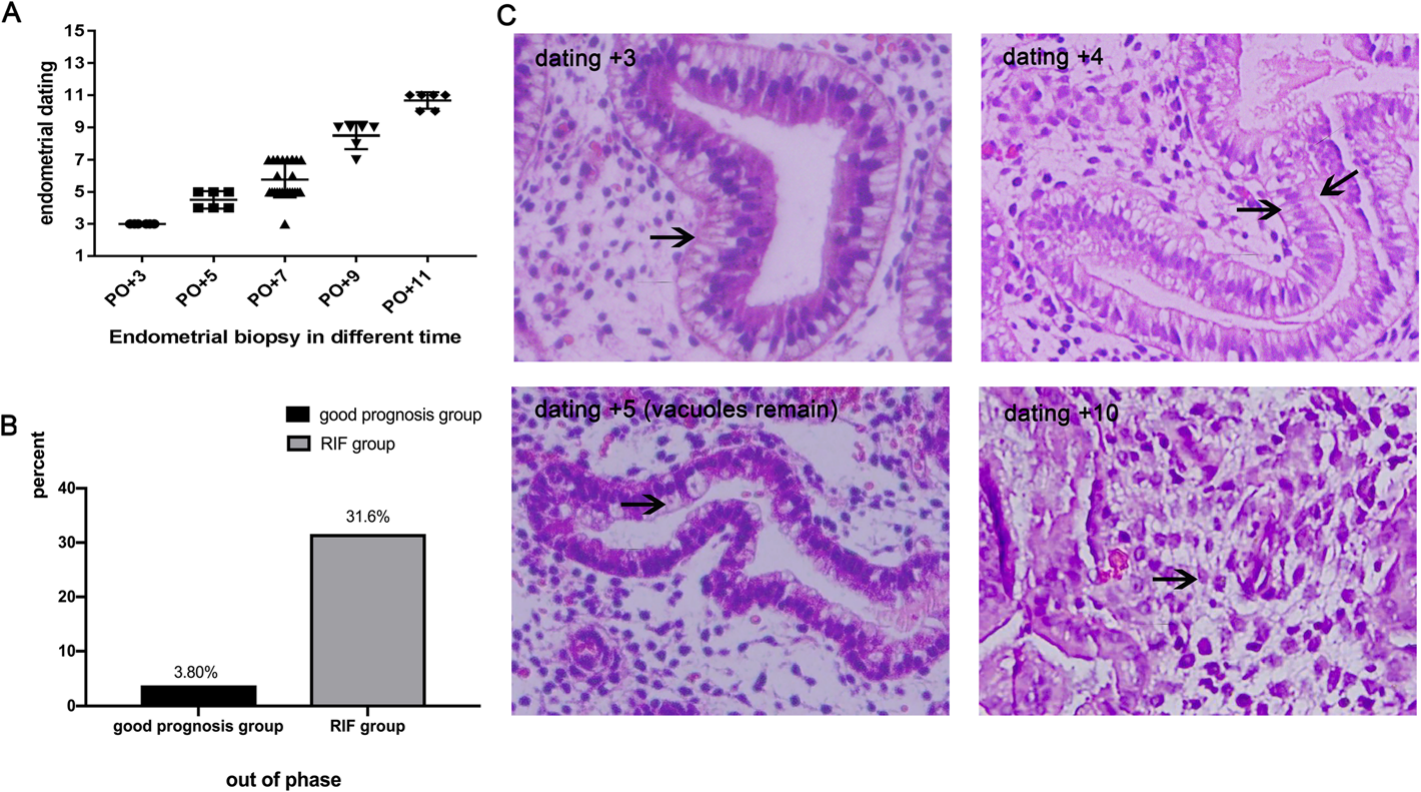
Endometrial dating in control and RIF groups. **a** Inner-group differences in endometrial biopsy dating in the control group at different times (PO + 3/5/7/9/11) were rare, as proven by two experienced pathologists. Using the mean ± SD as the lower and upper limits to define a reference range for endometrial biopsy dating, 1 of 50 control women were below the range. **b** The out-of-phase rate in the good-prognosis group was different from that for the RIF group. **c** Endometrial dating according to the Noyes criteria in RIF patients (X 400). Dating + 3, gland cytoplasm and nuclei above and vacuoles below (arrow); dating + 4, glandular nuclei are in the center of the cells and glycogen vacuoles are seen on two sides of the gland nuclei (arrow); dating + 5, vacuoles (arrow) remain in the basement membrane of the gland cells; dating + 10, pre-decidua (arrow) begin to differentiate and spiral arteries increase

Endometrial dating standards for different days (PO + 3/5/7/9/11) were established in good-prognosis patients who achieved an ongoing pregnancy in their first conventional FET cycle (*n* = 41) (Fig.1). Two experienced pathologists confirmed the endometrial dating using the Noyes criteria in the good-prognosis patient group, showing that endometrial dating on different PO days to be significantly different. In contrast, the inner-group differences were so small that the endometrial dating of most good-prognosis patients showed a mean ± SD between the lower and upper limits. The exception was only one endometrium of a good-prognosis patient that was biopsied on PO + 7, but was dated as PO + 3, and this patient was pregnant in a conventional FET cycle (Fig. 3a).

Blinded histologic dating of endometrial biopsies from RIF (*n* = 155) or good-prognosis patients (*n* = 26) before frozen-thawed embryo transfer was performed on day PO + 7. The rate of out-of-phase dating on day PO + 7 was significantly higher (31.6% vs. 3.8%, *P* = 0.003) in the RIF group relative to the good-prognosis group (Fig. 3b).

On day PO + 7, a total of 49 RIF patients were evaluated as being out of phase, and 106 RIF patients were assessed as being in phase. One third (*n* = 35) of in-phase patients were dated + 7, while the remainder (*n* = 71) of in-phase patients were dated + 5. In out-of-phase patients, 24% (*n* = 12) were dated + 3 and 73% (*n* = 36, Fig. 3c) were dated + 4 (Fig. 3c) or + 5 (vacuoles remained) (Fig. 3c), and one patient was diagnosed as dated PO + 10 (Fig. 3c).

### Clinical outcomes in RIF patients with endometrial dating results per pFET

The demographic characteristics and reproductive history of RIF patients, including age, body mass index, duration of infertility, and cause of infertility, are shown in Table 1. Previous failed cycles numbered 3.6 ± 0.7, with minimal and maximal values of 3 and 5 cycles, respectively. pFET was performed in 47 patients whose personal window of implantation (WOI) was delayed by 3 (*n* = 35) or 4 (*n* = 11) days, or advanced by 3 days (n = 1). Day-3 embryos or Day-5 blastocysts were then transferred using this strategy in natural cycles after 4 to 7 days of ovulation, resulting in a live-birth rate of 57.4% (27/47) in the first transfer attempt.

**Table 1:** Summary of the demographic characteristics, reproductive history and the clinical outcomes of RIF patients and control patients

	RIF patients (*n* = 155)	Good prognosis patients (*n* = 26)	*P* value
Age(y)	33.0 ± 3.7	29.1 ± 2.8	<0.001
Duration of infertility (year)	5.6 ± 2.6	3.2 ± 1.9	0.022
BMI (kg/m2)	20.9 ± 1.6	21.9 ± 2.3	<0.001
Basal FSH level (mIU/ml)	6.4 ± 2.7	5.6 ± 1.5	0.131
Endometrial thickness on the day of embryo transfer	11.5 ± 1.5	11.1 ± 2.2	0.263
Cause of infertility			0.367
Male factor	20/155(12.9%)	5/26 (19.3%)	
Tubal factor	135/155 (87.1%)	21/26 (80.7%)	
No. of previous failed cycles	3.6 ± 0.7	1	/
The out of phase rate	49/155 (31.6%)	1/26 (3.8%)	0.003
Total patients with 1st pFET/FET	47	26	
High quality embryo rate	33/47 (70.2%)	20/26 (76.9%)	0.793
Cleavage stage embryo	3/47 (6.4%)	3/26 (11.5%)	
Blastocyst	30/47 (63.8%)	17/26 (65.4%)	
Implantation rate after 1st pFET/FET	32/67 (47.8%)	23/41 (56.1%)	0.400
Ongoing pregnancies rate after 1st pFET/FET	29/47 (61.7%)	21/26 (80.7%)	0.093
Biochemical pregnancies after 1st pFET	7/47 (14.9%)	2/26 (7.6%)	0.476
live birth rate after 1st pFET/FET	27/47 (57.4%)	21/26 (80.7%)	0.07
Failed pregnancies after 1st pFET/FET	11	3	
No. of 2nd biopsies at the specified day	5	/	
2nd expectant endometrial dating	5	/	
Total patients with 2nd pFET/FET	5	2	
Implantation rate after 2nd pFET/FET	3/7 (42.8%)	0	
Ongoing pregnancies after 2nd pFET/FET	3/5 (60%)	0	
Accumulative live birth rate after pFET/FET	29/47 (61.7%)	21/26 (80.7%)	0.093

RIF patients who failed to become pregnant after the first pFET had a second endometrial biopsy delayed by 1–2 days according to the results of the first round of endometrial dating. All of these five patients then showed their expected endometrial dating result in their second endometrial biopsy. In the second pFET attempt, the live-birth rate was 40% (2/5), and thus the cumulative live-birth rate for personal FET was 61.7%.

## Discussion

Implantation is not a single event, but is more accurately described as a cascade of interactions between the embryo and endometrium. The human endometrium is receptive to embryonic implantation during a narrow period of the menstrual cycle referred to as the WOI. The WOI had been assumed to be constant for all women, although investigators have recently demonstrated the existence of a “displaced WOI” [15–18]. The classic method of dating the endometrium using defined histologic criteria was established in 1950 [8], but pFET studies in natural cycles using the Noyes criteria are presently lacking. In the present study, we established the endometrial dating criteria only for patients who became pregnant in their first conventional FET cycle. All our good-prognosis patients were pregnant, with histologic dating of days + 5–7 on day PO + 7, except for one patient whose histologic dating was PO + 3, which is consistent with the in-phase definition [9]. Endometrial dating was simultaneously evaluated by two experienced pathologists, and the inter-observer agreement was statistically determined to be acceptable. Thus, the endometrial dating criteria were easily mastered and utilized according to the Noyes criteria.

Finally, our data indicated that intra-group variation in the good-prognosis patients was so low that we considered our results to be highly reliable. We determined the reproducibility and verifiability of our endometrial dating results in the same patients, with five RIF patients who failed to become pregnant after the first pFET. These patients underwent a second endometrial biopsy delayed by 1 or 2 days according to the results of the first round of endometrial dating, and showed the expected endometrial dating results in the second endometrial biopsy. The second biopsies were conducted within 4 months, which suggested that the endometrial dating results might be repeatable in 4 months. Endometrial dating of a larger sample and longer period of time is still necessary to corroborate reproducibility. Displaced WOI may be detected by endometrial dating, and a subset of the patients with unexplained RIF may benefit from our study data.

This is the first pFET study to utilize endometrial dating verified by the Noyes criteria. First, we verified the Noyes criteria using endometrium samples from different time points from the pregnant good prognosis patients. Second, we compared the difference of the out-of-phase rate (displacement of WOI) between RIF patients and good-prognosis patients. Third, we determined individualized interventions (pFET) based on the verified criteria.

There were some limitations to our research. Although the results of previous donor oocyte IVF cycles suggested that recipients age ≤ 39 years had similar rates of implantation, clinical pregnancy, and live birth [19], we couldn’t exclude the impact of age on endometrial dating by recruiting younger female (20–35 years) patients for the good-prognosis group. The primary objective of our study was to use endometrial histology to detect WOI displacement in RIF patients, and to evaluate the clinical outcomes by pFET. However, there were no comparative data on clinical pregnancy rates or live-birth rates in women with RIF who had an adjusted FET, or on those who did not. We suggest that a randomized controlled study for RIF patients with out-of-phase dating be undertaken, and that further research be performed to determine the mechanism(s) underlying irregularities in the WOI.

## Conclusions

We observed an obviously increased percentage in WOI displacement in RIF patients compared with good-prognosis patients, leading us to propose pFET as a treatment strategy. FET personalized according histologic endometrial dating may improve the clinical outcomes of patients with unexplained RIF.

## Supplementary information

**Additional file 1: Figure S1.** Endometrial specimen dating according to Noyes criteria (X 400). A (dating + 3), gland nuclei were pushed to the center of the epithelial cells, with the cytoplasm above and vacuoles below (arrow). B (dating + 4), gland nuclei returned to the basilar side of the cells, and some vacuoles (arrow) were pushed past the nucleus to apparently empty glycogen into the lumen. C (dating + 5), few vacuoles remained, and the glandular cavity was filled with secretions (arrow). D (dating + 7), tissue edema. E (dating + 9), glands were highly distorted, jagged, or cauliflower-shaped (arrow). F (dating + 11), pre-decidua (arrow) began to differentiate under the surface epithelium.

## Data Availability

The datasets used and/or analyzed during the current study are available from the corresponding author upon reasonable request.

## Abbreviations

pFET: personalized frozen-thawed embryo transfer
RIF: repeated implantation failure
WOI: window of implantation
PO: post-ovulation
ET: embryo transfer
HRT: hormone-replacement cycle
IVF/ICSI: in vitro fertilization/intracytoplasmic sperm injection
ERA: endometrial receptivity array

## References

[1] Harton G, Braude P, Lashwood A, Schmutzler A, Traeger-Synodinos J, Wilton L, Harper JC, European Society for Human Reproduction and Embryology (ESHRE) PGD Consortium (2011). ESHRE PGD consortium best practice guidelines for organization of a PGD Centre for PGD/preimplantation genetic screening. Hum Reprod.

[2] Coughlan C, Ledger W, Wang Q, Liu F, Demirol A, Gurgan T, Cutting R, Ong K, Sallam H, Li TC (2014). Recurrent implantation failure: definition and management. Reprod BioMed Online.

[3] Minasi MG, Fiorentino F, Ruberti A, Biricik A, Cursio E, Cotroneo E, Varricchio MT, Surdo M, Spinella F, Greco E (2017). Genetic diseases and aneuploidies can be detected with a single blastocyst biopsy: a successful clinical approach. Hum Reprod.

[4] Achache H, Revel A (2006). Endometrial receptivity markers,the journey to successful embryo implantation. Hum Reprod Update.

[5] Aghajanova L, Hamilton AE, Giudice LC (2008). Uterine receptivity to human embryonic implantation: histology, biomarkers, and transcriptomics. Semin Cell Dev Biol.

[6] Nardo LG, Nikas G, Makrigiannakis A, Sinatra F, Nardo F (2003). Synchronous expression of pinopodes and alpha v beta 3 and alpha 4 beta 1 integrins in the endometrial surface epithelium of normally menstruating women during the implantation window. J Reprod Med.

[7] Lessey BA (2003). Two pathways of progesterone action in the human endometrium: implications for implantation and contraception. Steroids..

[8] Noyes RW, Hertig AI, Rock J (1950). Dating the endometrial biopsy. Fertil Steril.

[9] Wentz AC (1980). Endometrial biopsy in the evaluation of infertility. Fertil Steril.

[10] Sahmay S, Oral E, Saridogan E, Senturk L, Atasu T (1995). Endometrial biopsy findings in infertility: analysis of 12,949 cases. Int J Fertil Menopausal Stud.

[11] Zawar MP, Deshpande NM, Gadgil PA, Mahanta AA (2003). Histopathological study of endometrium in infertility. Indian J Pathol Microbiol.

[12] Coutifaris C, Myers ER, Guzick DS, Diamond MP, Carson SA, Legro RS (2004). Histological dating of timed endometrial biopsy tissue is not related to fertility status. Fertil Steril.

[13] Gomaa H, Casper RF, Esfandiari N, Bentov Y (2015). Non-synchronized endometrium and its correction in non-ovulatory cryopreserved embryo transfer cycles. Reprod BioMed Online.

[14] Kuwayama M (2007). Highly efficient vitrification for cryopreservation of human oocytes and embryos: the Cryotop method. Theriogenology.

[15] Lessey BA (2011). Assessment of endometrial receptivity. Fertil Steril.

[16] Galliano D, Bellver J, Díaz-García C, Simón C, Pellicer A (2015). ART and uterine pathology: how relevant is the maternal side for implantation?. Hum Reprod Update.

[17] Kliman HJ, Frankfurter D (2019). Clinical approach to recurrent implantation failure: evidence-based evaluation of the endometrium. Fertil Steril.

[18] Paulson RJ (2019). Introduction: endometrial receptivity: evaluation, induction and inhibition. Fertil Steril.

[19] Yeh JS, Steward RG, Dude AM, Shah AA, Goldfarb JM, Muasher SJ (2014). Pregnancy outcomes decline in recipients over age 44: an analysis of 27,959 fresh donor oocyte in vitro fertilization cycles from the Society for Assisted Reproductive Technology. Fertil Steril.

