# Supplementary figures and images for: Clinical value of histologic endometrial dating for personalized frozen-thawed embryo transfer in patients with repeated implantation failure in natural cycles
Source: BMC Pregnancy Childbirth. 2020 Sep 11;20:527. doi: 10.1186/s12884-020-03217-y (PMC7488450; doi:10.1186/s12884-020-03217-y)

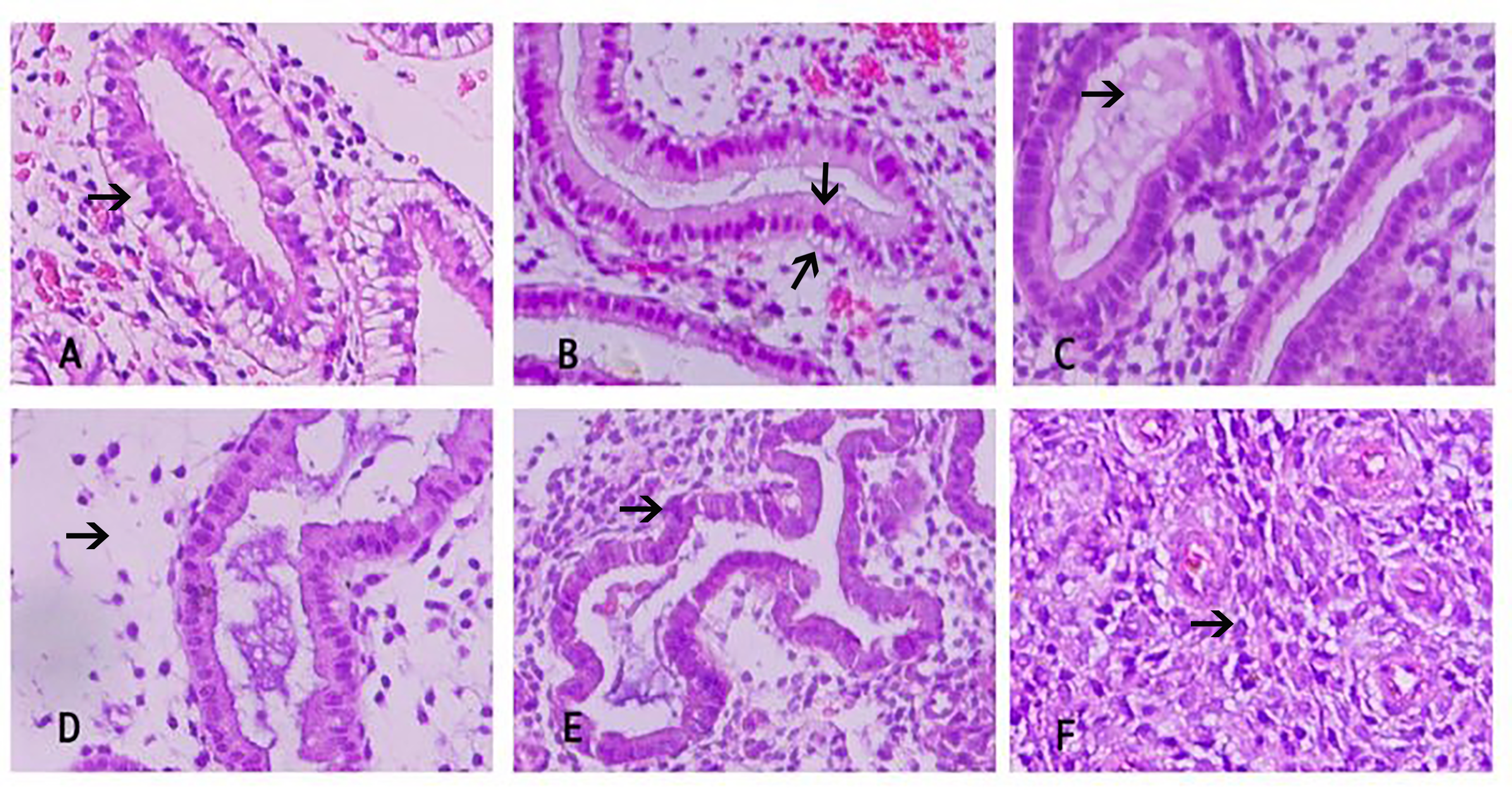

Supplement: Supplementary file 1 — Additional file 1: Figure S1. Endometrial specimen dating according to Noyes criteria (X 400). A (dating + 3), gland nuclei were pushed to the center of the epithelial cells, with the cytoplasm above and vacuoles below (arrow). B (dating + 4), gland nuclei returned to the basilar side of the cells, and some vacuoles (arrow) were pushed past the nucleus to apparently empty glycogen into the lumen. C (dating + 5), few vacuoles remained, and the glandular cavity was filled with secretions (arrow). D (dating + 7), tissue edema. E (dating + 9), glands were highly distorted, jagged, or cauliflower-shaped (arrow). F (dating + 11), pre-decidua (arrow) began to differentiate under the surface epithelium. [file 12884_2020_3217_MOESM1_ESM.tif]
